# Amelioration of Gamma-hexachlorocyclohexane (Lindane) induced renal toxicity by *Camellia sinensis* in Wistar rats

**DOI:** 10.14202/vetworld.2016.1331-1337

**Published:** 2016-11-30

**Authors:** W. L. N. V. Vara Prasad, Ch. Srilatha, N. Sailaja, N. K. B. Raju, N. Jayasree

**Affiliations:** 1 Department of Veterinary Pathology, College of Veterinary Science, Sri Venkateswara Veterinary University, Tirupati, Andhra Pradesh, India; 2 Department of Veterinary Pathology, College of Veterinary Science, Sri Venkateswara Veterinary University, Proddatur, Andhra Pradesh, India; 3 Department of Veterinary Anatomy, NTR College of Veterinary Science, Sri Venkateswara Veterinary University, Gannavaram, Andhra Pradesh, India

**Keywords:** *Camellia sinensis*, gamma-hexachlorocyclohexane, kidney

## Abstract

**Aim:**

A study to assess the toxic effects of gamma-hexachlorocyclohexane (γ-HCH) (lindane) and ameliorative effects of *Camellia sinensis* on renal system has been carried out in male Wistar rats.

**Materials and Methods:**

Four groups of rats with 18 each were maintained under standard laboratory hygienic conditions and provided feed and water *ad libitum*. γ-HCH was gavaged at 20 mg/kg b.wt. using olive oil as vehicle to Groups II. *C. sinensis* at 100 mg/kg b.wt. was administered orally in distilled water to Group IV in addition to γ-HCH 20 mg/kg b.wt. up to 45 days to study ameliorative effects. Groups I and III were treated with distilled water and *C. sinensis* (100 mg/kg b.wt.), respectively. Six rats from each group were sacrificed at fortnight intervals. Serum was collected for creatinine estimation. The kidney tissues were collected in chilled phosphate buffer saline for antioxidant profile and in also 10% buffered formalin for histopathological studies.

**Results:**

γ-HCH treatment significantly increased serum creatinine and significantly reduced the renal antioxidative enzymes catalase, superoxide dismutase, and glutathione peroxidase. Grossly, severe congestion was noticed in the kidneys. Microscopically, kidney revealed glomerular congestion, atrophy, intertubular hemorrhages, degenerative changes in tubular epithelium with vacuolated cytoplasm, desquamation of epithelium and urinary cast formation. A significant reduction in serum creatinine levels, significant improvement in renal antioxidant enzyme activities and near to normal histological appearance of kidneys in Group IV indicated that the green tea ameliorated the effects of γ-HCH, on renal toxicity.

**Conclusion:**

This study suggested that *C. sinensis* extract combined with γ-HCH could enhance antioxidant/detoxification system which consequently reduced the oxidative stress thus potentially reducing γ-HCH toxicity and tissue damage.

## Introduction

Lindane, the gamma-isomer of hexachlorocyclohexane (γ-HCH), is one of the oldest synthetic pesticides still in use worldwide. Lindane still is more commonly used to eradicate insects in agriculture and to treat lice infestation in humans, poultry, and livestock [1]. Its persistence in the environment, high mammalian toxicity, and resistance to biodegradation led to a ban or restricted use in many developed and developing countries, and this pesticide has become widely distributed in ecosystems and is now considered a global pollutant [2]. γ-HCH has a deteriorative effect on fauna and flora by inducing oxidative stress. γ-HCH is an inducer of the mixed function oxidase system such as cytochrome B_5_, nicotinamide adenine dinucleotide phosphate cytochrome P-450 reductase, and nicotinamide adenine dinucleotide - cytochrome C-reductase, which results in increased superoxide radical formation [3].

In India, the rich and diversified flora provides a valuable storehouse of medicinal plants. Homeopathic and phytotherapeutic remedies in veterinary medicine have gained an interest due to increasing demands on the quality of meat and milk products such as the requirements for producing good organic food [4]. *Camellia sinensis* commonly known as green tea is one of the most widely consumed beverages in the world, and many of its medicinal properties have been widely explored. Green tea polyphenols have demonstrated significant antioxidant [5], anticarcinogenic [6], and anti-inflammatory [7] properties.

Therefore, this study was aimed to evaluate a protective effect of green tea extract against γ-HCH induced renal damage by biochemical and histopathological analysis.

## Materials and Methods

### Ethical approval

The study protocol was approved from the Institutional Animal Ethics Committee constituted in accordance with the rules and guidelines of the Committee for the Purpose of Control and Supervision of Experiments and Animals, India (CPCSEA 722/06).

### Chemicals

Lindane (γ-isomer of HCH) was purchased from Sigma-Aldrich Pvt. Ltd., Bengaluru, India. The *C. sinensis* (green tea) whole leaf extract powder (methanolic extract containing 99.9% polyphenol) with product Code No. P/SVU/CASI-01 was procured from Chemiloids Company, Vijayawada, Andhra Pradesh state.

### Experimental animals

Male albino Wistar rats weighing between 150 and 200 g each were used for this experiment. They were procured from Sri Venkateswara Agencies, Bengaluru (REG NO: 237/99/CPCSEA). The rats were maintained in a controlled environment under standard conditions of temperature (28±2°C) and humidity with an alternating light and dark cycle. The rats were fed with commercially available pelleted rat chow and water *ad libitum*. After a week of acclimatization, rats were divided into control and test groups of 18 rats each. Lindane (20 mg/kg) dissolved in olive oil and green tea (100 mg/kg) dissolved in Water, were administered to rats (appropriate amounts dissolved in fixed volume of 0.3 ml) for 45 days by oral administration.

### Experimental design

The rats were divided into four groups of 18 rats in each. Group I served as control (0.3 ml of water by oral administration), Group II rats were treated with lindane (20 mg/kg b.wt. by oral administration), Group III rats were treated with green tea (100 mg/kg b.wt. dissolved in water by oral administration), Group IV rats were co-treated with lindane (20 mg/kg b.wt.) and green tea (100 mg/kg b.wt.), first the animals were treated with lindane followed by green tea 15 min later.

Six rats from each group were anesthetized by exposing to diethyl ether and then sacrificed by cervical decapitation. Blood was collected; serum was separated and used for renal marker assay. The kidney tissues were dissected out, washed in ice-cold saline, patted dry, and weighed. A small portion of the kidney tissue was stored in 10% formalin for histopathological examination. From the remaining tissue, about 100 mg tissue of kidney samples homogenized in chilled 0.1 M Tris-HCl buffer in Potter–Elvehjem Teflon homogenizer. The homogenates were used for biochemical investigation.

### Histopathology

A small portion of kidney tissue from the control and experimental animals was fixed in 10% neutral buffered formalin and processed by standard procedure for paraffin embedding and serial sections of about 5 μ size cut and were stained with hematoxylin and eosin.

### Measurement of creatinine

The activities of serum creatinine were estimated in blood serum using Prietest Robonik semi-automatic biochemical analyzer with commercial biochemical kits (Span Diagnostics). The results were expressed as mg/dl for serum creatinine.

### Measurement of antioxidants

The kidney tissue homogenates were used for the assay of superoxide dismutase (SOD), catalase (CAT), and glutathione peroxidase (GPx). CAT activity is determined by monitoring the decrease in absorbance spectrophotometrically at 240 nm due to decomposition of H_2_O_2_, the difference in extinction coefficient per unit time is a measure of the CAT activity [8]. Superoxide is an intermediate in the autooxidation of pyrogallol which occurs at pH 8.2, the ability of SOD to inhibit the autooxidation of pyrogallol at pH 8.2 provides the basis for enzymatic activity [9]. GPx reacts with H_2_O_2_ and reduced glutathione giving rise to oxidoreductase this will form a color complex with DTNB, the intensity of color development is directly proportional to amount of GPx present in the tissue [10]. The results were expressed as mg/g of protein for SOD, nM of H_2_O_2_ decomposed/min/mg of protein for CAT and μ of glutathione utilized/min/mg protein for GPx.

### Statistical analysis

The values for all parameters were expressed as mean±standard error for six rats sacrificed from each group at fortnight intervals. Data were analyzed using one-way analysis of variance [11].

## Results

**Figure-1:**
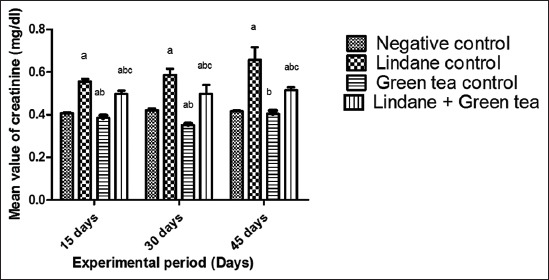
Mean values of serum creatinine (mg/dl) in rats of diffe rent experimental groups. (n=18) Values bearing different superscripts in the figure are significantly different (p<0.05).

Mean serum creatinine values of γ-HCH treated group (Group II) significantly (p<0.05) increased compared to control group (Group I) from 15^th^ to 45^th^ day of the experiment. Green tea ameliorated rats (Group IV) showed significant (p<0.05) reduction in serum creatinine compared to Group II from 15^th^ to 45^th^ day of the experiment. Green tea ameliorated rats (Group IV) showed significantly (p<0.05) higher serum creatinine levels than control group from 15^th^ to 45^th^ day of the experiment. Green tea treated group (Group III) showed significant (p<0.05) reduction in serum creatinine compared to the control group (Figure-1) from 15^th^ to 45^th^ day of the experiment.

A significant (p<0.05) reduction was observed in mean renal SOD, CAT and GPx activity from 15^th^ to day 45 of γ-HCH treated rats (Group II) compared to the control group (Group I). Green tea ameliorated rats (Group IV) showed significant (p<0.05) increase in renal antioxidative enzymes compared to Group II from 15^th^ to 45^th^ day of experiment (Table-1).

**Table 1:** The ameliorative effect of *C. sinensis* on γ-HCH induced changes in renal antioxidants (n=18).

Biochemical parameters	Days at sacrifice	Group I	Group II	Group III	Group IV
SOD (mg/g of protein)	15^th^ day (Mean±SD)	16.62±0.132	11.20±0.09^a^	16.01±0.29^ab^	16.00±0.26^ab^
	30^th^ day (Mean±SD)	17.00±0.34	9.85±0.73a	15.90±0.07^ab^	15.28±0.35^ab^
	45^th^ day (Mean±SD)	16.5.0±0.22	7.42±0.086^a^	16.90±0.063^b^	14.05±0.96^abc^
CAT (nM of H_2_O_2_ decomposed/min/mg of protein)	15^th^ day (Mean±SD)	0.200±0.020	0.170±0.0124^a^	0.220±0.014^b^	0.200±0.006^b^
	30^th^day (Mean±SD)	0.223±0.010	0.135±0.008^a^	0.220±0.008^b^	0.180±0.0.008^abc^
	45^th^ day (Mean±SD)	0.210±0.014	0.075±0.032^a^	0.210±0.008^b^	0.180±0.006^abc^
GPx (μ of glutathione utilized/min/mg protein)	15^th^ day (Mean±SD)	28.01±1.86	24.50±2.31^a^	26.40±1.01	25.40±2.42
	30^th^ day (Mean±SD)	27.50±0.68	18.40±1.49^a^	27.90±1.35^b^	23.01±2.62^abc^
	45^th^ day (Mean±SD)	28.20±0.56	13.20±2.43^a^	28.24±0.64^b^	20.51±1.30^abc^

^a^represents ^a^superscript bearing group significantly differ from Group I (p<0.001), ^b^represents ^b^superscript bearing group significantly differ from Group II (p<0.001), ^c^represents ^c^superscript bearing group significantly differ from Group III (p<0.001), Mean values with different superscripts differ significantly (p<0.05). ANOVA, SD=Standard deviation, *C. sinensis*=*Camellia sinensis*, γ-HCH=γ-hexachlorocyclohexane, SOD=Superoxide dismutase, CAT=Catalase, GPx=Glutathione peroxidase

**Figure-2:**
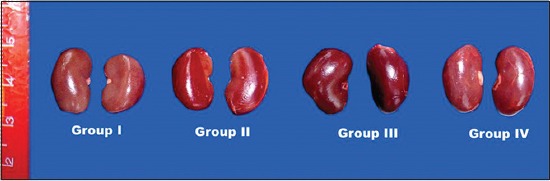
Congestion of gamma hexachlorocyclohexane treated rats kidney compared to other groups 45^th^ day of experiment.

Grossly, there were no observable lesions in kidneys of rats during the 15^th^ day of the experiment but severe congestion of kidneys was noticed by the end of the 30^th^ and 45^th^ day (Figure-2), and these changes were absent in the kidneys of the ameliorated group.

**Figure-3:**
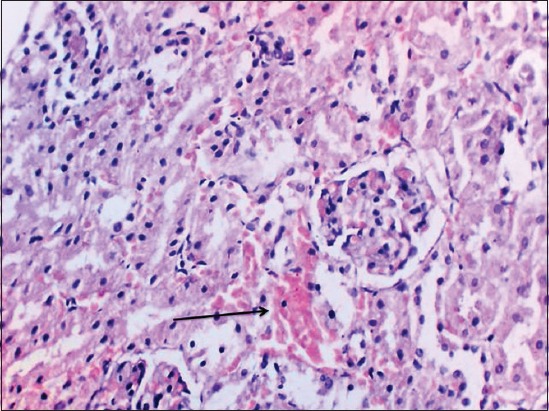
Degeneration of renal tubular epithelial cells and intertubular haemorrhages in kidney of gamma hexachlorocyclohexane treated rats 15^th^ day of experiment (H and E, 400×).

Kidney of γ-HCH treated animals at the end of the 15^th^ day of the experiment revealed congestion, mild glomerular congestion, and intertubular hemorrhages. Mild degenerative changes were noticed in the tubular epithelium (Figure-3) with swollen cells, condensed nuclei and loss of brush borders in proximal convoluted tubules and reduced lumen.

**Figure-4:**
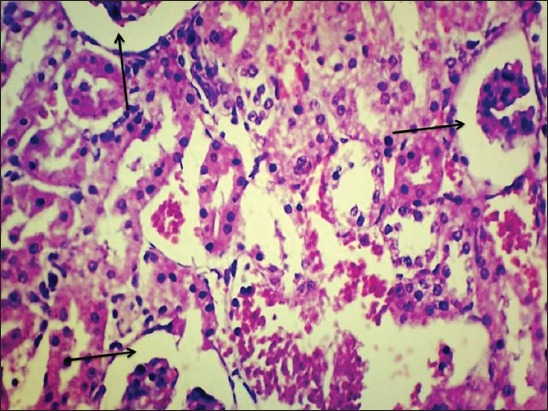
Increased bowman capsular spaces in kidney of gamma hexachlorocyclohexane treated rats 30^th^ day of experiment (H and E, 400×).

**Figure-5:**
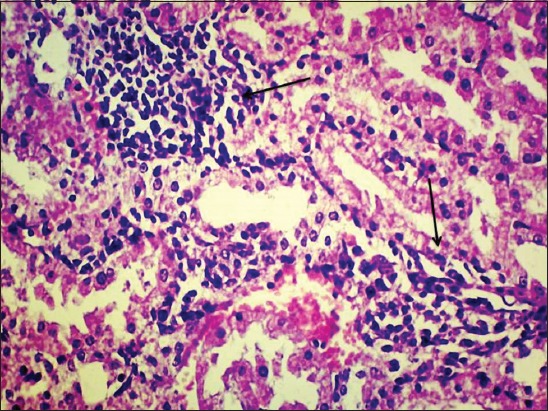
Severe degenerative changes in tubular epithelium and diffuse infiltration of mononuclear cell in kidney of gamma hexachlorocyclohexane treated rats 30^th^ day of experiment (H and E, 400×).

By the end of the 30^th^ day, kidneys of Group II rats revealed severe intertubular hemorrhages, increased bowman’s capsular space in glomeruli (Figure-4), cystic dilatation of tubules, severe degenerative changes in tubular epithelium, diffuse infiltration of mononuclear cell (MNC) (Figure-5), and microgranuloma formation followed by necrosis and desquamation into lumen and cast formation.

**Figure-6:**
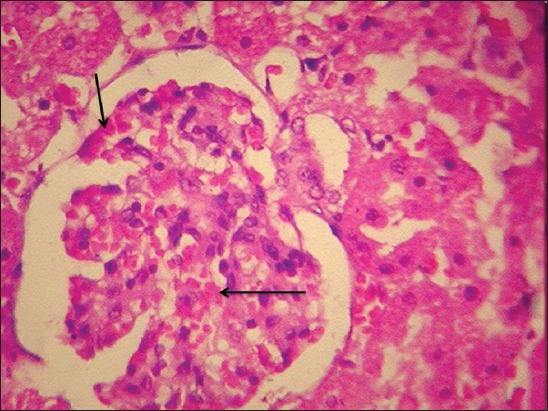
Glomerular congestion in kidney of gamma hexachlorocyclohexane treated rats 45^th^ day of experiment (H and E, 400×).

**Figure-7:**
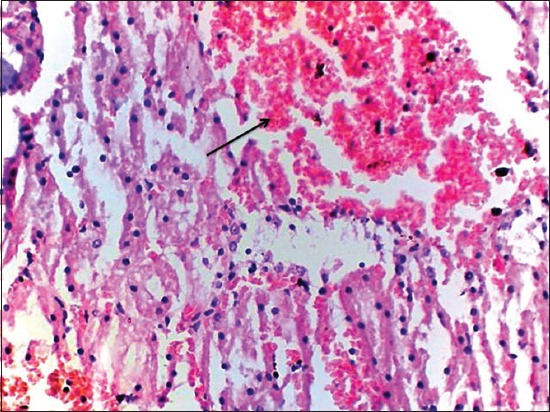
Severe intertubular hemorrhages in kidney of gamma hexachlorocyclohexane treated rats 45^th^ day of experiment (H and E, 400×).

**Figure-8:**
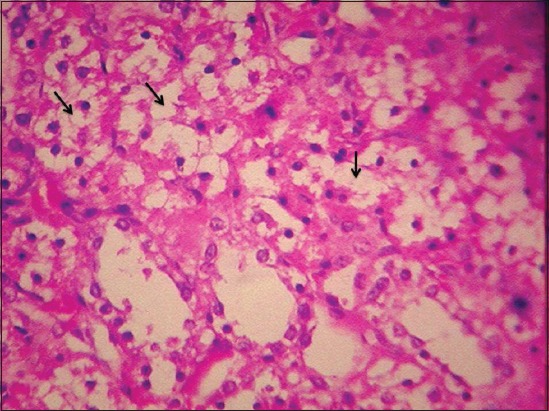
Severe degenerative changes in tubular epithelium with vacuolated cytoplasm in kidney of gamma hexachlorocyclohexane treated rats 45^th^ day of experiment (H and E, 400×).

**Figure-9:**
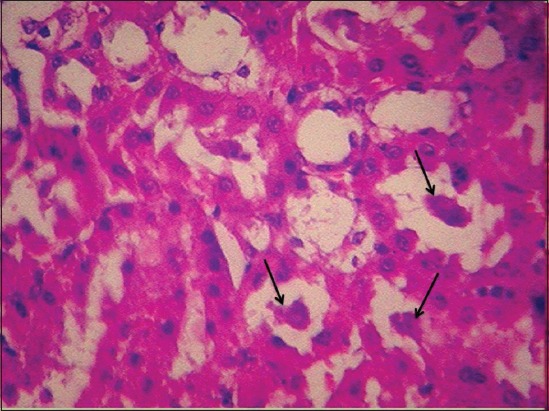
Severe desquamation of tubular epithelium leading to cast formation in kidney of gamma-hexachlorocyclohexane treated rats 45^th^ day of experiment (H and E, 400×).

**Figure-10:**
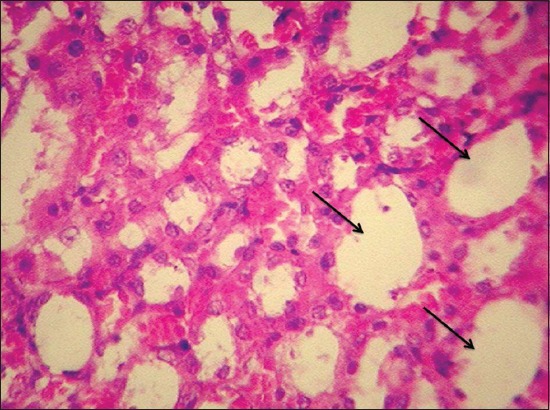
Cystic dilatation of tubules in kidney of gamma-hexachlorocyclohexane treated rats 45^th^ day of experiment (H and E, 400×).

**Figure-11:**
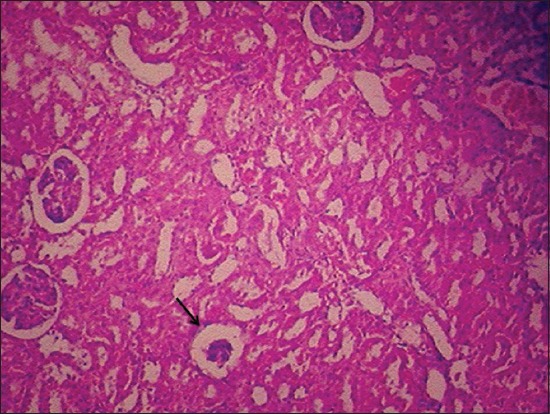
Atrophic glomeruli and necrotic changes in renal tubular epithelium of g hexachlorocyclohexane treated rats 45^th^ day of experiment (H and E, 100×).

At the end of the 45^th^ day, kidneys of Group II rats revealed severe corticomedullary congestion, glomerular congestion (Figure-6), and intertubular hemorrhages (Figure-7). Severe degenerative changes in tubular epithelium with vacuolated cytoplasm (Figure-8) and desquamation of tubular epithelium leading to cast formation (Figure-9) and cystic dilatation of tubules (Figure-10) and necrosis were observed. Atrophic glomeruli and diffused areas of MNC infiltration were also observed (Figure-11).

**Figure-12:**
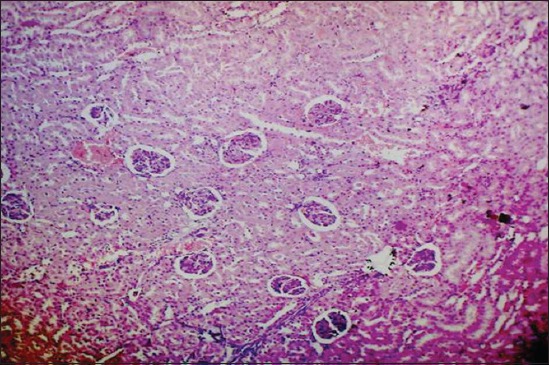
Kidney regained to its near to normal appearance in green tea treated rats by 30^th^ day of experiment (H and E, 100×).

**Figure-13:**
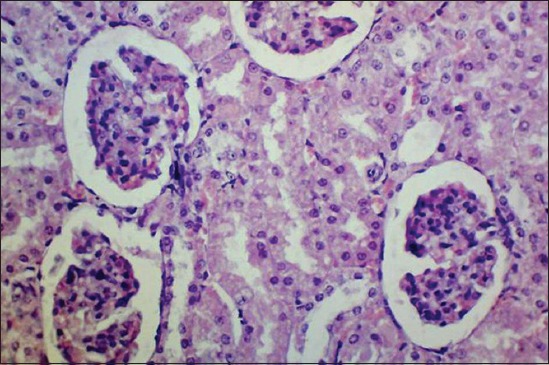
Kidney regained to its near to normal appearance in green tea treated rats by 45^th^ day of experiment (H and E, 400×).

Kidney sections of the green tea ameliorated rats revealed similar lesions as that of Group II till the end of the 15^th^ day. However, the 30^th^ day, the congested glomerulus, mild intertubular hemorrhages and mild degenerative changes in renal tubular epithelial cells (Figure-12) were noticed. Almost near to normal appearance of kidneys was evident in all the animals by the end of the 45^th^ day (Figure-13).

## Discussion

Serum creatinine levels of γ-HCH treated rats showed significant increase when compared to the control rats. Similar reports were made by previous author [12,13] according to them serum concentration of endogenous creatinine was used as a measure of renal function because it is an easily measured by product of muscle metabolism that is excreted unchanged by the kidneys. The increased levels of serum creatinine in γ-HCH treated rats might be due to alteration in renal metabolism [14]. Significantly increased level of creatinine was observed in γ-HCH treated rats and is supported by renal damage evident microscopically and it might be due to oxidative stress induced by free radical interaction with the cell membrane, triggering the generation of reactive oxygen species (ROS) and other free radical intermediates and altering the levels of antioxidant molecules or enzymes such as CAT, SOD, and GPx [2]. Green tea ameliorated group showed significant decrease in serum creatinine values compared to γ-HCH treated group but failed to reach the levels in control group rats. This is in accordance to earlier reports [15,16], who reported that green tea has polyphenols which have antioxidant property, scavenge a wide range of free radicals. Polyphenols prevent the metal catalyzed formation of radical species, antioxidant enzyme modulators, prevents oxidative damage and might have prevented the rise of serum creatinine levels.

A significant decrease in renal CAT, SOD and GPx was observed in γ-HCH treated rats when compared to control group rats from the 2^nd^ week and continued up to the end of the experiment. These findings are in line with previous report [12]. The decrease in antioxidant enzyme activity might be due to γ-HCH induced generation of ROS [17] which further leads to oxidative damage of kidney and these were evidenced microscopically in the present study. A significant increase in CAT, SOD and GPx activities was noticed in ameliorated group when compared to γ-HCH treated rats, and the results are in accordance with previous authors [15,16,18]. This increase might be because of antioxidant activity of green tea. In this study, ameliorated group of rats showed significantly lower CAT, SOD and GPx activity than control group in all weeks indicated the amelioration to a certain extent though not completely ameliorating the toxic effect. These protective effects of green tea were also supported by histological restoration of normal renal architecture.

There was no significant change in kidneys of rats during the 15^th^ day of experiment but severe congestion of kidneys was noticed by the end of the 30^th^ and 45^th^ day, and these changes were absent in the kidneys of ameliorated group. These were in accordance to earlier reports [19].

Kidney revealed congested corticomedullary blood vessels, glomerular congestion, and intertubular hemorrhages microscopically. Degenerative changes in tubular epithelium with swollen cells, condensed nuclei, and loss of brush borders in proximal convoluted tubule were observed throughout the experiment. Increased bowman’s capsular space in glomeruli, desquamation of renal tubular epithelium and urinary cast formation and diffuse intertubular infiltration of inflammatory cells were prominent in addition to the above changes in the 30^th^ and 45^th^ day of the experiment. Similar findings were reported by earlier authors [16,19]. Renal oxidative stress in this study is evidenced by a significant reduction in renal antioxidant enzymes CAT, SOD, and GPx. Creatinine which is considered as a renal marker is also significantly increased following γ-HCH administration. Histopathological examination of kidney of green tea ameliorated groups exhibited similar lesions but in milder form in some of the rats, but kidney regained its near to normal appearance by the end of the experiment. This might be due to antioxidant and free radical scavenging property of polyphenols of *C. sinensis* [15,16]. These protective effects of green tea were also supported by restoration of serum creatine levels and renal antioxidant enzymes.

## Conclusion

The accumulation of γ-HCH in the kidneys was associated with oxidative stress and tissue injury as detected by histopathology. The treatment of rats with *C. sinensis* extract combined with γ-HCH could enhance antioxidant/detoxification system which consequently reduced the oxidative stress. The beneficial effect of *C. sinensis* in improving antioxidant status was associated with reduction of γ-HCH burden in kidneys thus potentially reducing γ-HCH toxicity and tissue damage.

## Authors’ Contributions

ChS, NS and NKBR planned, guided and supervised the entire research work. WLNVVP carried out the experimental work, laboratory analysis, histopathological analysis and data analysis. WLNVVP drafted the first manuscript with the help of NJ. ChS and NS revised the manuscript. All authors read and approved the final manuscript.
